# Early transduction produces highly functional chimeric antigen receptor-modified virus-specific T-cells with central memory markers: a Production Assistant for Cell Therapy (PACT) translational application

**DOI:** 10.1186/s40425-015-0049-1

**Published:** 2015-02-18

**Authors:** Jiali Sun, Leslie E Huye, Natalia Lapteva, Maksim Mamonkin, Manasa Hiregange, Brandon Ballard, Olga Dakhova, Darshana Raghavan, April G Durett, Serena K Perna, Bilal Omer, Lisa A Rollins, Ann M Leen, Juan F Vera, Gianpietro Dotti, Adrian P Gee, Malcolm K Brenner, Douglas G Myers, Cliona M Rooney

**Affiliations:** Center for Cell and Gene Therapy Baylor College of Medicine Texas Children’s Hospital Houston Methodist Hospital, Houston, TX 77030 USA; Department of Pathology and Immunology, Baylor College of Medicine, Houston, TX 77030 USA; Department of Pediatrics, Baylor College of Medicine, Houston, TX 77030 USA; Department of Medicine, Baylor College of Medicine, Houston, TX 77030 USA; Children’s Mercy Hospitals and Clinics, Kansas City, MO 64108 USA; Department of Molecular Virology and Immunology, Baylor College of Medicine, Houston, TX 77030 USA

**Keywords:** Virus-specific T cells, Chimeric antigen receptor, Clinical grade T-cell manufacture

## Abstract

**Background:**

Virus-specific T-cells (VSTs) proliferate exponentially after adoptive transfer into hematopoietic stem cell transplant (HSCT) recipients, eliminate virus infections, then persist and provide long-term protection from viral disease. If VSTs behaved similarly when modified with tumor-specific chimeric antigen receptors (CARs), they should have potent anti-tumor activity. This theory was evaluated by Cruz et al. in a previous clinical trial with CD19.CAR-modified VSTs, but there was little apparent expansion of these cells in patients. In that study, VSTs were gene-modified on day 19 of culture and we hypothesized that by this time, sufficient T-cell differentiation may have occurred to limit the subsequent proliferative capacity of the transduced T-cells. To facilitate the clinical testing of this hypothesis in a project supported by the NHLBI-PACT mechanism, we developed and optimized a good manufacturing practices (GMP) compliant method for the early transduction of VSTs directed to Epstein-Barr virus (EBV), Adenovirus (AdV) and cytomegalovirus (CMV) using a CAR directed to the tumor-associated antigen disialoganglioside (GD2).

**Results:**

Ad-CMVpp65-transduced EBV-LCLs effectively stimulated VSTs directed to all three viruses (triVSTs). Transduction efficiency on day three was increased in the presence of cytokines and high-speed centrifugation of retroviral supernatant onto retronectin-coated plates, so that under optimal conditions up to 88% of tetramer-positive VSTs expressed the GD2.CAR. The average transduction efficiency of early-and late transduced VSTs was 55 ± 4% and 22 ± 5% respectively, and early-transduced VSTs maintained higher frequencies of T cells with central memory or intermediate memory phenotypes. Early-transduced VSTs also had higher proliferative capacity and produced higher levels of T_H_1 cytokines IL-2, TNF-α, IFN-γ, MIP-1α, MIP-1β and other cytokines *in vitro*.

**Conclusions:**

We developed a rapid and GMP compliant method for the early transduction of multivirus-specific T-cells that allowed stable expression of high levels of a tumor directed CAR. Since a proportion of early-transduced CAR-VSTs had a central memory phenotype, they should expand and persist *in vivo*, simultaneously protecting against infection and targeting residual malignancy. This manufacturing strategy is currently under clinical investigation in patients receiving allogeneic HSCT for relapsed neuroblastoma and B-cell malignancies (NCT01460901 using a GD2.CAR and NCT00840853 using a CD19.CAR).

**Electronic supplementary material:**

The online version of this article (doi:10.1186/s40425-015-0049-1) contains supplementary material, which is available to authorized users.

## Background

A major problem with chimeric antigen receptor (CAR)-modified T-cells for the treatment of solid tumors is their lack of *in vivo* proliferation [1,2]. Even when costimulatory endodomains are incorporated into CARs, CAR-T-cells may fail to proliferate in the presence of immunosuppressive tumors that not only lack costimulatory ligands but actively inhibit T-cell proliferation by expressing inhibitory ligands, such as PD-L1 and secreting inhibitory cytokines such as TGF-β [3-5]. By contrast to tumors, viruses are highly immunostimulatory and T-cells with native TCR specificity for viruses (VSTs) proliferate exponentially after infusion into HSCT recipients because patients are lymphopenic and viruses are poorly controlled, increasing the abundance of viral antigens [6]. We reasoned that if VSTs were engrafted with tumor-specific CARs, then extratumoral stimulation by endogenous viruses would ensure CAR-T-cell expansion *in vivo* and might even restore the function of T-cells anergized by the tumor. Hence CAR-VSTs could both protect against viral infections after HSCT and eliminate residual tumor.

In a previous clinical trial we tested the hypothesis that extratumoral stimulation by an endogenous virus would ensure CAR-T-cell expansion *in vivo* in children with relapsed neuroblastoma infused with autologous EBV-specific T-cells (EBVSTs) genetically modified to express a CAR specific for GD2, a disialoganglioside that is highly expressed by this tumor [1,2]. We expected that endogenous EBV would provide *in vivo* stimulation of GD2.CAR-modified EBVSTs, increasing their expansion and anti-tumor function relative to similarly-transduced CD3-activated T-cells (GD2.CAR-ATCs). In this original study, each T-cell component expressed a GD2.CAR that differed only in a few non-coding nucleotides that allowed us to compare the fate of infused GD2.CAR-ATCs and GD2.CAR-EBVSTs in each patient treated. This combination of T-cells was clinically effective, producing tumor responses in 5 of 11 patients and complete responses in three. However, although transduced EBVSTs were detected at higher levels than transduced ATCs in the six weeks following infection, they did not apparently expand in numbers, at least as measured in the circulation, and tumor responses were associated with the long-term persistence of either population, albeit at low levels. Hence it was unclear which population was responsible for the clinical responses.

As an National Heart, Lung, and Blood Institute (NHLBI)-funded Production Assistance for Cell Therapies (PACT) site, we were charged with the production of donor-derived T cells specific for EBV, CMV and adenovirus (triVSTs) transduced with the first generation GD2.CAR, for pediatric patients receiving haploidentical HSCT for the treatment of relapsed neuroblastoma at the Children’s Mercy Hospital, Kansas City, MO (Principle Investigator Dr. GD Myers, NCT01460901). In this new protocol, the intent was to determine if infusion of GD2.CAR-triVSTs after T-cell depleted HSCT could overcome the previous lack of expansion by providing a lymphopenic environment in which homeostatic cytokines are in excess and viruses are poorly controlled and therefore more likely to stimulate CAR-modified VSTs. The use of T-cells specific for three viruses rather than one should increase the chances that T-cells would be stimulated after HSCT, since CMV, EBV and adenoviruses commonly, but not always coincidentally, reactivate after HSCT. We proposed that several modifications to the GD2.CAR-modified VST generation protocol would also improve the ability of the modified T-cells to expand and persist in recipients.

In the previous study [1], EBVSTs were generated by stimulation of PBMCs with irradiated, autologous EBV-transformed B lymphoblastoid cell lines (EBV-LCLs). IL-2, the cytokine used for EBVST expansion, was not introduced until day 13 to ensure EBV specificity and optimal transduction efficacy (40% to 50%), could not be achieved until day 19 of culture (late transduction) at which time the rate of T-cell proliferation was at its peak. However, by this time significant *in vitro* differentiation had occurred with loss of T-cells with an early-differentiated phenotype (CD45RO^+^ CCR7^+^, central memory cells and CD45RO^+^, CCR7^−^, CD62L^+^, T-cells with an intermediate phenotype), while most T-cells had an effector memory (CD45RO^+^ CCR7^−^, CD62L^−^) phenotype. Moreover, after transduction, at least 11 days of additional expansion were required to obtain sufficient cells for infusion, and during this time, the frequency of transduced T-cells often decreased, while the population continued to differentiate. This prolonged 30-day *ex vivo* culture may have adversely impacted subsequent T cell proliferation *in vivo*. These issues may explain the lack of *in vivo* proliferation we observed in a recent clinical trial that evaluated triVSTs transduced with a second generation CD19.CAR for the treatment of patients with lymphoma after allogeneic HSCT [7]. In this study, T-cells were also transduced after their third antigenic stimulation on day 19. We proposed that lack of *in vivo* expansion could result from late transduction of VSTs with insufficient proliferative potential.

Here we describe a new strategy for the early retroviral transduction of minimally differentiated triVSTs that produces high and stable levels of transgene expression in all three VST components and allows rapid expansion to numbers sufficient for infusion within 8 to 16 days of culture.

## Results

For the new study we wished to transduce VSTs with a less differentiated, preferentially central memory phenotype in the hope that they would have greater potential to proliferate after infusion*.* We hypothesized that by increasing the potency of the initial stimulation we would be able to transduce VSTs after their first stimulation (early transduction) and cryopreserve them for infusion on day 8 to 16 of culture versus day 30 or later for late transduced VSTs. Further, to transduce T-cells with specificity for three viruses we had to be sure that our stimulation produced sufficient stimulation of all populations with similar kinetics. Hence, *ex vivo* measure of success would be (1) high levels of transduction of VSTs with specificities for all three viruses (EBV, CMV and adenovirus), (2) stable gene expression over two to three *in vitro* stimulation cycles, (3) sufficient transduced VSTs for our clinical use by day 16 of culture, (4) transduced VSTs with a central memory phenotype.

### EBV-specific T-cells (EBVSTs) can be transduced effectively on day 3 of culture

**Figure 1:**
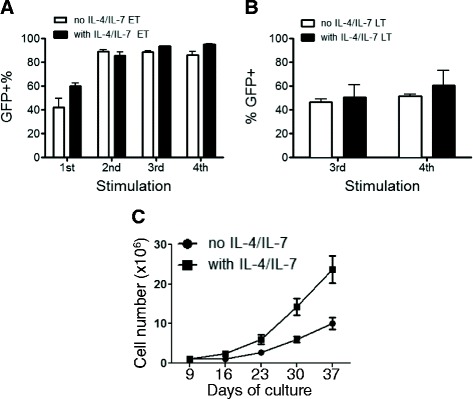
**Optimization of early transduction of EBVSTs with a retroviral vector encoding GFP.** PBMCs were stimulated with autologous EBV-LCLs with or without IL-4 and IL-7. Two days later, the cells were transduced with a high titer retroviral vector encoding GFP. The transgene (GFP) expression by EBVSTs transduced **(A)** on day 2 (ET) or **(B)** on day 19 (LT) was determined by FACS analysis after the number of stimulations indicated. (**C**) Show the total cell numbers of Day 2 (ET) transduced cells in the presence or absence of IL-4 and IL-7 over time. Data represent the mean +/− sd of 2 donors.

To enhance the transduction efficiency of VSTs on day 2 or day 3 we attempted to increase the potency of the first PBMC stimulation without loss of virus specificity. In pilot experiments, we used a high-titer GFP-encoding retroviral vector to transduce EBVSTs. The transduction efficiency was similar when measured 4 days after transduction (about 40%) in PBMCs transduced 3 days after their first stimulation with EBV-LCLs (early transduction, ET) (Figure 1A) or 3 days after their third stimulation on day 19 (late transduction, LT) (Figure 1B). However, the transgene frequency in early transduced EBVSTs increased to more than 80% by day 14 after transduction, reflecting the specific expansion of the EBV-specific component of the culture and loss of irrelevant PBMCs. By contrast, the frequency of transduced T-cells in LT cultures showed little change following subsequent stimulations; likely because there is little change in the frequency of EBVSTs after the third stimulation.

### Proliferation of transduced EBVSTs can be increased without loss of viral specificity by stimulation in the presence of IL-4 and IL-7

We have previously shown that the addition of the prosurvival cytokines IL-4 and IL-7 (IL-4/7) during the first stimulation of VSTs increases their rate of expansion and broadens their TCR repertoire, in part by increasing their expression of anti-apoptotic molecules like bcl2 and bcl_XL_ [8]. Furthermore, IL4/7-grown VSTs have been tested in two clinical trials and were successfully able to resolve viral infections after HSCT [9,10]. We therefore evaluated the effect of IL-4/7 added during the first stimulation of PBMCs and at the time of transduction. While the initial transduction efficiency when measured on day 4 was not substantially affected by IL-4/7 (Figure 1A and B), there was greater expansion of early-transduced EBVSTs cultured with IL-4/7 than without cytokines (Figure 1C), potentially allowing us to achieve clinically relevant numbers of transduced EBVSTs more rapidly.

**Figure 2:**
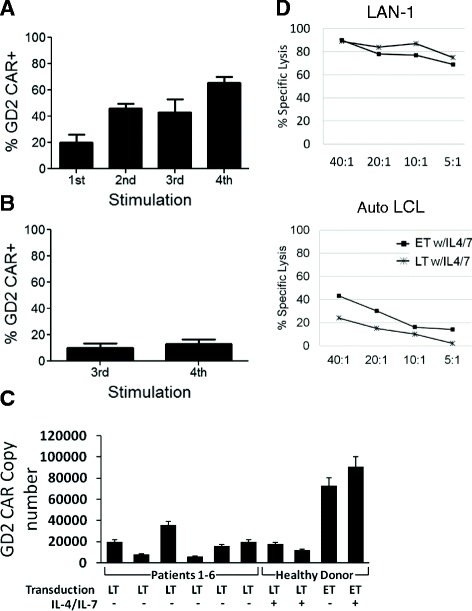
**Early transduction of EBVSTs with GD2.CAR vector.** PBMCs were stimulated four times with irradiated autologous LCLs on days 0, 9, 16 and 23 of culture. They were transduced with the GD2.CAR retroviral vector either on day 3 or day 19. GD2.CAR transgene levels were measured by flow cytometry on **(A)** day 3- early transduced (ET) or **(B)** day 19 late-transduced (LT) EBVSTs at the end of each stimulation. Data represent the mean +/− sd of 2 donors. **(C)** The transduction efficiency of ET or LT cells was compared with patient-derived EBVSTs (transduced on day ~19) from the previous clinical trial [1] using real-time PCR analysis for the retroviral vector. Day 19-transduced (LT) EBVSTs and patient lines demonstrated similar transduction efficiencies while Day 3-transduced (ET) EBVSTs attained a much higher transgene expression. Transgene PCR was performed 5 to 8 days post-transduction. **(D)** Specific killing was determined in a 6-hour ^51^Chromium-release assay using the GD2 expressing neuroblastoma cell line, LAN-1 (top), and autologous LCL (bottom).

Having established that EBVSTs can be transduced efficiently after their first stimulation with a high titer GFP control retroviral vector, we next explored early transduction of EBVSTs with the less efficient, clinical grade first generation GD2.CAR retroviral vector that was to be used in the proposed clinical trial. In these experiments transduction efficiency was higher in EBVSTs transduced on day 3 (mean 20%; range 15.94% to 24.5%) (Figure 2A) than in EBVSTs transduced on day 19 (mean 10.1%; range 7.73% to 12.5%) (Figure 2B). Furthermore, the increase in the frequency of transduced cells in day 3-transduced EBVSTs to mean of 66% (range 63% to 69%) upon subsequent stimulations was not observed in day 19-transduced EBVSTs (mean 13%; range 6.6% to 19.6%).

To validate these phenotypic measures of transduction efficiencies, we used RT-PCR 5 to 8 days post-transduction to measure the integrated retroviral copy numbers and compared these transduction efficiencies to those achieved in the patient-derived EBVSTs used in our earlier clinical trial [1]. Day 19 transduced EBVSTs and the patient EBVSTs exhibited similar GD2.CAR transduction efficiencies (14,816 copies ± 4191 copies and 17,450 ± 10,632, per μg DNA respectively), while day 3-transduced EBVSTs had much higher GD2.CAR transduction efficiencies (81,983 copies ± 12,572 copies per μg DNA) (Figure 2C). Consistent with their expression of the GD2.CAR, both early and late-transduced EBVSTs killed GD2-positive LAN1 neuroblastoma cells, whilst retaining their ability to kill autologous EBV-LCLs through their MHC-restricted native TCRs (Figure 2D).

### Early transduced T cells have a less differentiated phenotype than late-transduced T-cells

**Figure 3:**
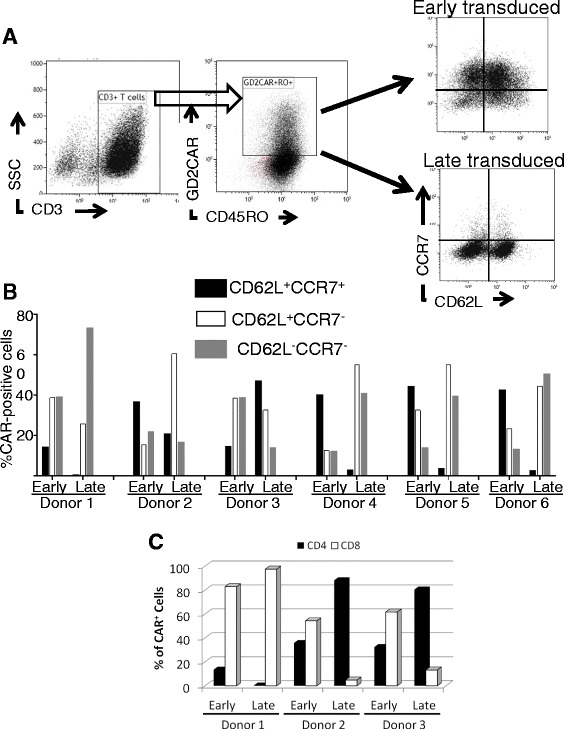
**The effector memory phenotype of T** **-** **cells transduced on day 3 and day 19.** The phenotype of Day 3 (early) and Day 19 (late)-transduced VSTs was determined by flow cytometry after staining for CD3, CD45RO, CD62L, CCR7, CD8 and the GD2.CAR. **(A)** Gating of T cell memory subsets. CD3^+^GD2.CAR^+^ CD45RO^+^ cells were gated and their memory phenotype classified based on differential expression of CD62L and CCR7. Representative dot-plots from one donor out of six are shown **(B)**. Percentages of CD62L^+^CCR7^+^ central memory cells, CD62L^+^CCR7^−^ intermediate, CD62L^−^CCR7^−^ effector memory T cells were gated as above in six donors. **(C)** The frequency of CD4^+^ and CD8^+^ cells in the CD3^+^GD2.CAR^+^ gate. The analysis of the phenotype was performed at the time of potential cryopreservation (day 11 for Day 3 transduced VSTs and day 26 for Day 19 transduced VSTs).

Since VSTs transduced on day 3 can potentially be cryopreserved for infusion at the end of their first (days 9 to 11) or second (day 16 to 18) proliferative cycles, they are likely to be less differentiated than VSTs transduced on day 19, after their third stimulation and cryopreserved for infusion at the earliest on day 26. Therefore, we analyzed Day 3- and Day 19-transduced VSTs for markers of T-cell differentiation (CD45RO, CD62L, and CCR7) to determine the differentiation state of the CAR-positive VSTs at the time of cryopreservation (day 11 for Day 3-transduced VSTs and day 26 for Day 19-transduced VSTs). In 5 of 6 donors, day 3-transduced VSTs contained a higher percentage of central memory cells expressing both CD62L and CCR7 (Figure 3A and B), supporting our hypothesis that early transduction enables transduction of a cell product with a less differentiated state and likely greater memory potential. Of note, day 3 transduction of VSTs produced cultures in which both CD4^+^ and CD8^+^ VSTs were transduced, while transducing on Day 19 sometimes favored the transduction of either CD4^+^ or CD8^+^ cells(Figure 3C).

### Simultaneous induction of trivirus-specific T-cells (triVSTs)

**Figure 4:**
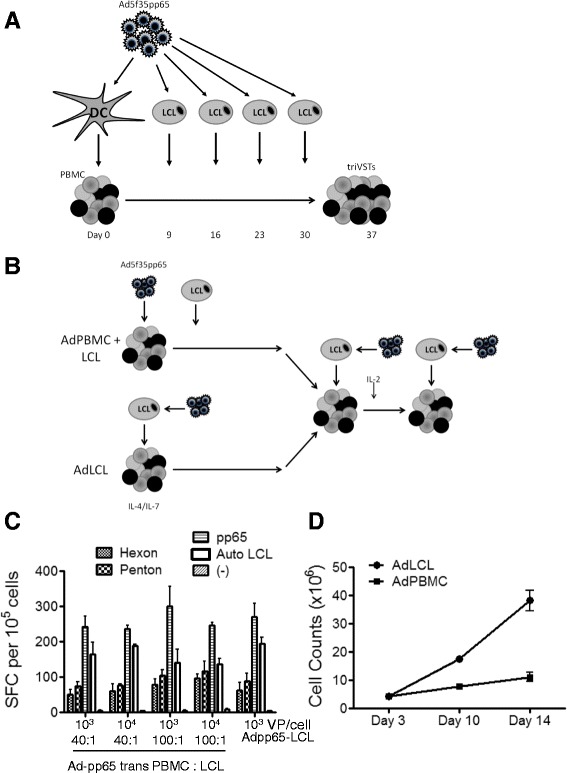
**Simultaneous induction of trivirus** **-** **specific T** **-** **cells. (A)** A diagram depicting the standard protocol for the generation of triVSTs. **(B)** Comparison of two protocols for the simultaneous induction of triVSTs. In Ad/PBMC + LCL protocol, PBMCs were cultured in non-tissue culture treated plates overnight then transduced with Ad5f35-pp65 and cocultured with autologous LCLs. In the Ad/LCL protocol, LCLs were transduced with the Ad5f35-pp65 vector and used for autologous PBMC stimulation. **(C)** Day 9 after the first stimulation, the AdV (hexon and penton), CMV (pp65) and EBV (Auto LCL) specificities were determined by ELIspot assay using pepmixes for hexon, penton and pp65 and autologous LCLs for EBV. The MOI of Ad5f35-pp65 transduction and the ratio of PBMC to LCL are indicated. **(D)** TriVSTs were generated using Ad/LCLs and Ad/PBMC + LCLs (100:1) and counted on days 3, 10, and 14. Data represent the mean +/− sd of 2 donors.

The proposed clinical trial called for the preparation of GD2.CAR-transduced T-cells specific for CMV, EBV and adenovirus. Although we had previously generated (non-transduced) triVSTs for clinical use [11], the first stimulation of PBMCs was with Ad5f35-pp65-transduced monocytes or dendritic cells that provided CMV pp65 as a transgene and processed and presented adenovirus hexon and penton from the virion, EBV antigens were not introduced until day 9, when the VSTs were restimulated with Ad5f35-transduced EBV-LCLs (Figure 4A). Early transduction of triVSTs requires that PBMCs be stimulated with similar kinetics and potency with antigens from all three viruses. Therefore, we compared two methods of presenting antigens from all three viruses to PBMCs from CMV and EBV-seropositive donors. In the first method, PBMCs were adhered to plastic overnight to activate monocytes then were transduced with Ad5F35-pp65 and cocultured with EBV-LCLs (Ad/PBMC + LCL method Figure 4B, top). In the second method, EBV-LCLs were transduced with Ad5F35-pp65, irradiated and cocultured with PBMCs at a stimulator to responder ratios of (40:1) (Ad/LCL method Figure 4B, bottom). For the Ad/PBMC + LCL method, we tested different multiplicities of infection (MOI) of virus particle (vp) per cell ratios (10^3^ and 10^4^ vp per cell) and different Ad/PBMC:LCL ratios (40:1 and 100:1), and found that all conditions induced T-cells specific for all three viruses and there was little difference between any of the conditions (Figure 4C). T-cells specific for all three viruses were also activated using the Ad/LCL method (Figure 4C), which induced greater expansion of triVSTs than the Ad/PBMC + LCL method (Figure 4D). Since this method was simpler than the Ad/PBMCs + LCL method, we elected to use the Ad/LCL method for our clinical standard operating procedure (SOP).

**Figure 5:**
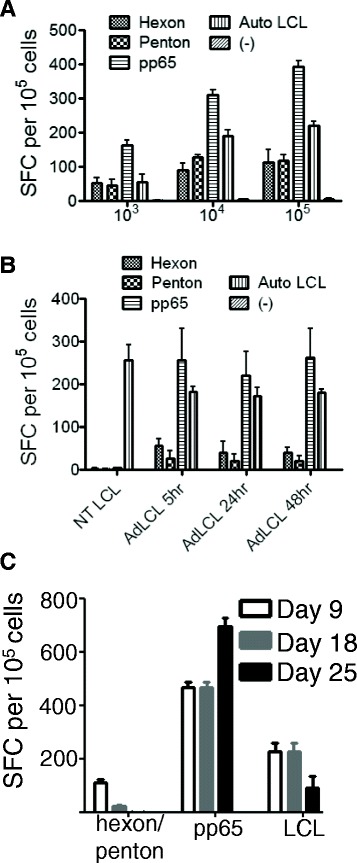
**Optimization of the induction of trivirus specificity with Ad5f35** **-** **pp65 transduced LCL. (A)** Trivirus specificity was determined by γ-IFN ELIspot assay 9 days after the 1st stimulation. The MOIs of Ad5f35-pp65 transduction of LCLs were 10^3^, 10^4^, and 10^5^ vp per cell respectively. **(B)** LCLs were transduced with an MOI of 10^4^ and cultured for the indicated times before being irradiated and used for PBMC stimulation. ELIspot assays were performed 9 days after the 1st stimulation. Non-transduced (NT) LCLs were used as controls. Data represent the mean +/− sd of 2 donors. **(C)** Tri-virus specific CTLs were initiated on Day 0 and re-stimulated on Days 9 and 18. Tri-virus specificities were assessed by γ-IFN ELIspot assay with LCLs or overlapping libraries of pepmixes spanning adenovirus hexon and penton and CMV pp65 proteins.

We further optimized Ad5f35-pp65 transduction of EBV-LCLs by comparing Ad5f35 MOIs of 10^3^, 10^4^, and 10^5^. MOI’s of 10^4^ and 10^5^ vps per cell produced higher T-cell responses to all three viruses than the MOI of 10^3^ (Figure 5A) and since there was little difference between the two higher ratios, an MOI of 10^4^ was used for the clinical standard operating procedure (SOP). Since we were concerned that the different kinetics of antigen presentation of the pp65 transgene, the virion proteins of the vector and the pre-existing EBV genes would lead to competition between the different epitopes on the antigen presenting LCLs, we also explored the optimum time for Ad5f35-pp65 transduction of LCLs prior to their use as APCs. However, we found little difference whether they were transduced 5 hours, 24 hours or 48 hours prior to coculture with PBMCs (Figure 5B). Therefore, for convenience, 24 hours was selected for our clinical SOP. As in our previous study, the fraction of T-cells specific for CMV-pp65 was greater than for adenovirus or EBV, reflecting the generally higher precursor frequency of CMV-specific T-cells in circulation [12,13].

We also found that the prolonged culture of tri-virus specific T cells led to skewing responses to more dominant antigens, such as pp65 (Figure 5C) compared to EBV and adenovirus. This is clear after the third stimulation (day 25), when adenovirus and EBV-specific responses were diminished. Therefore early transduction and cryopreservation of tri-VSTs after one or two stimulations allow balanced specificity for all three viruses.

### T-cells specific for CMV, adenovirus and EBV are similarly transduced with the GD2.CAR vector

**Figure 6:**
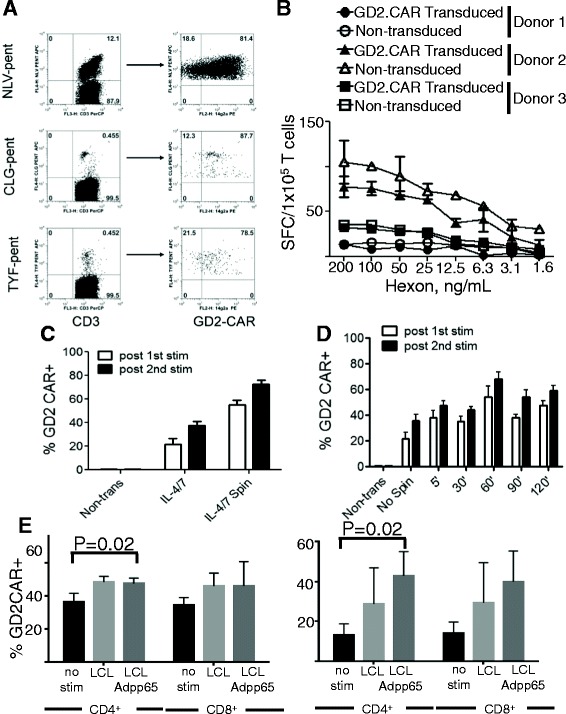
**Improving the efficiency of the TriVST transduction. (A)** PBMCs were stimulated using the Ad/LCL protocol in the presence of IL-4 and IL-7 and transduced on day 3 with the GD2.CAR vector. Co-expression of the GD2.CAR on pentamer positive CMV (A2-NLV), EBV (A2-CLG) and AdV (A24-TYF)-specific T-cells was analyzed by flow cytometry after the 3rd stimulation to confirm the transduction of triVSTs. The dot plots of one representative donor are shown. **(B)** GD2.CAR-transduced and non-transduced triVSTs from three donors were analyzed in IFN-γ ELISPOT with serial dilutions of overlapping pepmix library spanning adenovirus hexon. Responses were analyzed by two-way ANOVA and no statistical significant difference (p > 0.5) between transduced and non-transduced cells were found **(C)** The transduction efficiency with or without centrifugation of the retrovirus vector at 2000 × G onto the retronectin-coated plates is shown. **(D)** The transduction efficiency of VSTs after the indicated centrifugation time for the retroviral supernatant. Data represent the mean +/− sd of 2 donors. **(E)** Surface expression levels of GD2.CAR were upregulated in early transduced (ET) CD4^+^ and CD8^+^ T cells (left panel) and in late transduced (LT, right panel) triVSTs upon stimulation with LCLs and LCLs transduced with Ad5f35-pp65 (LCL Adpp65) for 48 hours without cytokines. Results are shown as mean ± SD, n = 3.

To ensure that each virus-specific component of the triVSTs elicited by Ad-LCLs could be transduced efficiently, PBMCs stimulated with Ad5f35-pp65-transduced LCLs in the presence of IL-4 and IL-7 were transduced on day 2 and then analyzed for dual specificity for a viral epitope and the GD2-CAR by flow cytometry. The triVST line shown in Figure 6A had specificity for all three viruses detectable by pentamer analysis (Figure 6A left panel). The pentamer positive populations were gated and analyzed for their expression of the GD2.CAR. Figure 6A (right panel) shows the coexpression of the GD2.CAR on 81% of CMV (HLA A2-NLV) pentamer + T-cells, 88% of EBV (HLA A2-CLG) pentamer-positive T-cells, and 79% of AdV (HLA A24-TYF)-pentamer positive VSTs.

To confirm that endogenous TCRs in CAR-transduced VSTs are functional and remain sensitive to stimulation with cognate antigens, we tested GD2.CAR-transduced and non-transduced T cells from three donors in IFN-γ ELISPOT assays with serial dilutions of peptide libraries (pepmixes) spanning hexon, pp65 and LMP2 proteins. There was no significant difference (p > 0.05) between responses in non-transduced and CAR-VSTs (Figure 6B and Additional file 1: Figure S1). Further, there was no significant difference (p > 0.05) in frequency (99.3% ± 0.4% in CAR-transduced VSTs and 99.4% ± 0.4%, n = 3) of αβTCRs in GD2.CAR transduced and non-transduced cells from three donors.

### VST transduction efficiencies can be increased by centrifugation of retroviral supernatant onto retronectin-coated plates

We next determined if centrifugation of viral supernatant onto retronectin-coated plates for two hours at 2000 × G as recommended by Takara Bio, would improve the transduction efficiency of triVSTs. After the centrifugation, the retroviral supernatant was removed prior to plating the triVSTs in medium with cytokines. The centrifugation step significantly increased transduction efficiency from mean of 22 (range 18% to 25%) to mean of 55% (range 52% to 58%) (Figure 6C) and the centrifugation time could be reduced to one hour without loss of transduction efficiency (Figure 6D). Of note, the removal of the retroviral supernatant after centrifugation increased the rate of expansion of VSTs after transduction, without compromising the transduction efficiency (not shown), likely reflecting removal of inhibitory factors present in the crude supernatant.

### CAR expression is increased by TCR stimulation

While GD2.CAR was stable in early transduced T-cells over time, the percentage of CAR^+^ cells increased in response to TCR stimulation after 48 hours of culture without cytokines in both CD4^+^ and CD8^+^ populations of early and late-transduced triVSTs (Figure 6E and Additional file 1: Figure S2). The upregulation of CAR was more profound in late-transduced cells as early-transduced VSTs were likely more activated and thus had higher basal expression of the transgene. This is consistent with our previous observations on enrichment of CAR-expressing EBVSTs upon stimulation with LCLs [14] and further strengthens the rationale for using VSTs as hosts for tumor-specific CARs.

### Early transduced triVSTs produce multiple cytokines and have higher proliferative capacity than late-transduced triVSTs

**Figure 7:**
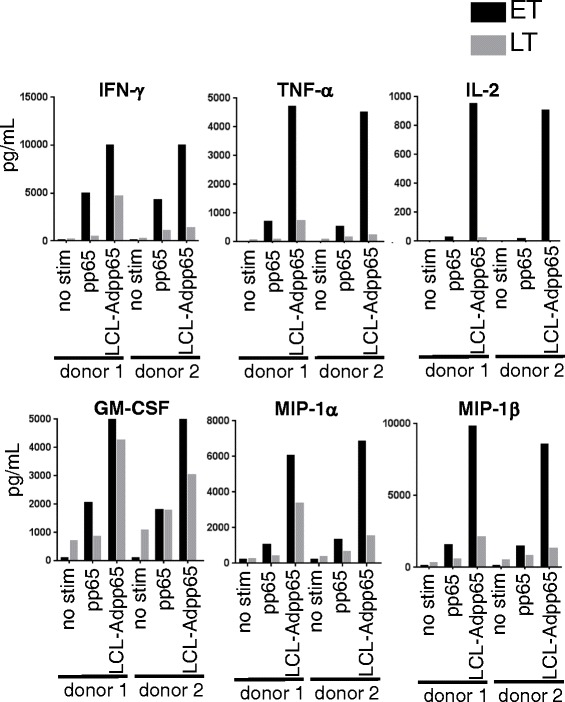
**Cytokines predominantly produced by early differentiated triVSTs.** One million early (ET) or late (LT) transduced triVSTs from two donors were stimulated with the pp65 pepmix or 2.5×10^5^ autologous LCLs transduced with Ad5f35-pp65. Supernatants were collected 24 hours after the stimulation and analyzed for multiple cytokines using multiplex cytokine magnetic bead assay.

We compared cytokine production by early and late-transduced VSTs after stimulation with a pp65 pepmix and Ad-pp65 transduced LCLs for 24 hours in a cytokine multiplex assay. Early-transduced VSTs produced higher levels of IFN-γ, TNFα, IL-2, GM-CSF, MIP-1α, MIP-1β, IL-3, IL-5, IL-8, IL-10, IL-13, IFN-α2, fractalkine, VEGF and sCD40L (Figure 7 and data not shown). By contrast, late transduced triVSTs produced higher levels of TNF-β and chemokines CXCL10 (IP-10) and CCL22 (MDC) (Additional file 1: Figure S3).

**Figure 8:**
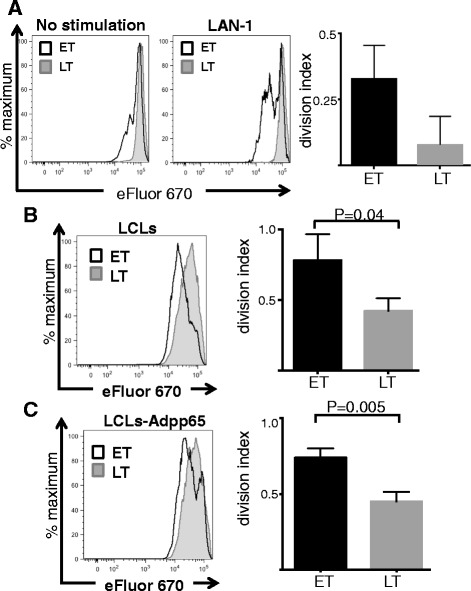
**Improved proliferation of early transduced triVSTs.** Early transduced (ET) and late transduced (LT) triVST with retrovirus expressing GD2.CAR were co-cultured for 72 hours with **(A)** GD2-positive LAN-1 neuroblastoma cells, or with **(B)** autologous LCLs at 4:1 of VST to LCL ratio or **(C)** Ad5f35-pp65-transduced LCLs (LCL-Adpp65). Proliferation of CD3^+^ T cells was evaluated by eFluor dilution by flow cytometry. Histograms are representative results from one donor. Cell division indexes were calculated using FlowJo software. Results are shown as mean ± SD, n = 3.

To compare the proliferative potential of early and late-transduced T-cells we performed eFluor dilution assays in CAR-triVSTs co-cultured with the GD2-positive neuroblastoma cell line LAN-1, LCLs and LCLs-transduced with Ad-pp65. Figure 8 shows greater proliferation of early-transduced VSTs than late-transduced VSTs in response to both CAR and TCR stimulation. CD8^+^ early-transduced VSTs proliferated significantly more (p = 0.0067) in response to LCLs compared to late-transduced VSTs (Additional file 1: Figure S4). Together these data suggest that the function and proliferative potential of early transduced VSTs may be greater than that of late-transduced VSTs.

### Validation of GMP-compliant GD2.CAR-modified triVST production

**Figure 9:**
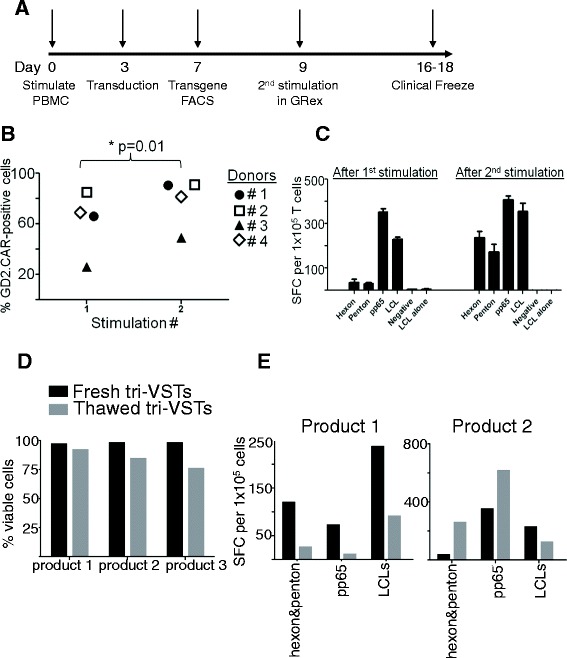
**GMP validation of the SOP for early transduction of TriVSTs. (A)** Diagram of the finalized SOP. Day 0, PBMC were stimulated with irradiated, Ad5f35-transduced autologous LCLs and then transduced on day 3. On day 9, triVSTs were harvested and restimulated in a G-Rex100 gas permeable flask. Cryopreservation was performed after the second stimulation on days 16–18 of culture. **(B)** Transgene expression after the 1st and 2nd stimulation was determined by flow cytometry in 4 donors. **(C)** Trivirus specificity was determined by γ-IFN ELIspot assay after the 1st and 2nd stimulations. 1x10^5^ responder cells per well were pulsed with overlapping peptide libraries for hexon, penton (to test for adenovirus specificity) and pp65 (to test for CMV specificity) and LCLs (to test for EBV specificity). One representative donor of three is shown. **(D)** Three clinical products of CAR-transduced triVSTs were cryopreserved in 10% DMSO, 12.5% HSA (Flexbumin, Baxter) and 1xHBSS in a Cryomed controlled-rate freezer. Viability of fresh and thawed cells was evaluated using trypan blue exclusion method. **(E)** INF-γ ELISPOT assay results for fresh and thawed CAR-transduced tri-VSTs. Adenovirus-specific responses were evaluated using pepmix libraries spanning hexon and penton, CMV with a pepmix library for pp65 and EBV-specific responses were analyzed using autologous LCLs.

Finally, to validate our optimized procedure in our cGMP facility, PBMCs from blood bank-eligible donors known to be seropositive for all three viruses were stimulated at a PBMC to Ad/LCL ratio of 40:1 on Day 0 at an MOI of 10^4^ vp per cell and supplemented with 400 U/mL IL-4 and 10 ng/mL IL-7 (IL-4/7). On day 3 of culture T cells were transduced with the GD2.CAR retroviral vector in the presence of fresh IL-4 and IL-7. Transduced triVSTs were harvested on Day 9 and restimulated at a 5:1 ratio of Ad5f35-pp65-transduced LCLs:VSTs in a gas permeable G-Rex flask with IL-4 and IL-7. IL-2 (50 U/ml) was added after the second stimulation every 3 to 4 days starting on day 12 and the cells were cryopreserved on day 16–18 when sufficient triVSTs for potential clinical use was obtained. During the first stimulation, we obtained 5.4 ± 1.8-fold (N = 3) T cell expansion. For example, starting with 24 × 10^6^ PBMCs, we obtained 173 × 10^6^ triVSTs on day 8. Transduced triVSTs expanded by 178 ± 110-fold during the second stimulation, increasing from 2 × 10^6^ on day 8 to 231 × 10^6^ on day 16. The average total expansion during two stimulations was 961.2-fold. Since triVST precursors constitute only a small fraction of total PBMCs, the true fold expansion of VSTs was likely at least 100-fold greater than reflected by the bulk expansion. A schematic of the validation run is shown in Figure 9A. Of note, since very small numbers of VSTs (2 × 10^7^ triVSTs per m^2^) are required in the HSCT setting, sufficient GD2.CAR-modified triVSTs were obtained on day 9 for potential clinical use.

During the validation runs, transgene expression increased from 61 ± 25% (mean ± sd) after the first stimulation to 78 ± 20% after the second stimulation (Figure 9B). T-cells specific for all three viruses were detected by IFN-γ ELISPOT assay after both the first and second stimulations (Figure 9C). Of note, slightly greater expansion and a higher transduction efficiencies were obtained in the validation runs, compared to research expansions, likely reflecting the modified medium formulation used in our GMP facility in which RPMI-1640 was replaced with Advanced RPMI. The average viability of infusion-grade triVSTs at the time of cryopreservation was 97.7% ± 0.6% (n = 3) (Figure 9D), which was greater than the 70% release criterion for clinical products. To evaluate potential viability and function after infusion, CAR-modified triVSTs were thawed (viability was 84.2% ± 8%) and cultured overnight before testing specificity in IFN-γ ELISPOT assays (Figure 9E). All products retained their responses to adenovirus (hexon and penton pepmixes), CMV (pp65) and EBV (LCLs), although in two donors the responses were lower than in fresh cells (Figure 9E left panel and data not shown) while in the other, responses to adenovirus proteins and pp65 were augmented (Figure 9E right panel) in thawed cells.

## Discussion

VSTs have the potential to proliferate extensively after infusion into T-cell-depleted HSCT recipients and to control the viral infections to which these patients are susceptible [9,10,15,16]. If these proliferating VSTs express a transgenic tumor-specific CAR as well as their TCR, then viruses should be able to increase their frequency, even if the tumors targeted by the CAR are poorly immunogenic or immunosuppressive. With the ultimate goal of producing CAR-modified VSTs with a central memory phenotype and hence the potential to proliferate extensively after infusion and to persist long term we have developed and optimized a GMP compliant strategy for the early transduction of triVSTs. Stimulation of PBMCs with irradiated, autologous LCLs transduced with an adenovirus vector expressing the pp65 protein of CMV allowed the presentation of antigens from all three viruses with kinetics that enabled their simultaneous transduction on day 3 as demonstrated by the expression of a GD2.CAR on pentamer-positive T-cells. Transduction on day 3 and the addition of prosurvival cytokines allowed rapid expansion and early cryopreservation of GD2.CAR-modfied triVSTs for use in a clinical trial in patients who have received T-cell depleted HSCT for multiply relapsed neuroblastoma. On day 3 of culture, VSTs with a central memory phenotype could be transduced, and CD45RO^+^ CCR7^+^ CD62L^+^ central memory transduced VSTs remained in the cultures at the end of the first stimulation. Early-transduced T-cells should have a greater potential to proliferate, eliminate tumors, and enter the memory compartment than VSTs transduced on day 19 at which time the majority of cells have lost expression of CCR7 and have an effector memory phenotype. Hence, we propose that GD2.CAR-modified T-cells specific for CMV, EBV and adenovirus should be effective both for the prevention and treatment of the three most common viral infections of HSCT recipients and for the treatment of neuroblastoma.

It is clear that the anti-tumor efficacy of adoptively-transferred tumor-specific T-cells requires extensive *in vivo* proliferation and long-term persistence since tumor eradication may take several weeks or months. It is also important that infused T-cells enter the memory compartment so that they can prevent the outgrowth of dormant tumors over the patient’s life-time. Such proliferation requires potent *in vivo* stimulation, not only with antigen via the CAR or the TCR, but also via costimulatory receptors and cytokines. However, even if they have not downregulated HLA antigens, most tumors do not present antigens in an immunostimulatory fashion. They lack costimulatory molecules and/or express inhibitory ligands and cytokines that inhibit T-cells and subvert the functions of local professional APCs. Therefore infused tumor-specific T-cells are unlikely to receive adequate stimulation after infusion. CD19.CAR T-cells, which have been particularly successful in the clinic [17,18], may be an exception since the B-cells they target are professional antigen presenting cells and express costimulatory molecules, as do normal B-cells that are also targets for CD19.CARs. Even when costimulatory domains are included in the CAR so that transduced cells receive intrinsic costimulation upon ligation of the CAR, cytokines are usually lacking, and the T-cells frequently fail to proliferate extensively. Cytokines can be infused, but are toxic and can enhance the proliferation of T regulatory cells [19]. Consequently, most groups use cytotoxic lymphodepletion to promote the subsequent homeostatic proliferation of adoptively transferred CAR.T-cells [18].

By contrast to tumors, most viral infections are highly immunostimulatory, since not only are viral antigens presented to the immune system, but viral structures such as virion proteins and viral nucleic acids provide pattern-associated molecular patterns (PAMPs) that activate innate immune responses that coordinate and amplify the adaptive immune response. After T-cell-depleted HSCT, the recipient is lymphopenic so that homeostatic cytokines are abundant and endogenous viruses frequently reactivate providing the ideal conditions for VST expansion. However, even after HSCT, in the absence of active infection or virus reactivation, little VST expansion may occur and we have observed minimal expansion of adenovirus-specific T-cells in the absence of adenovirus infection, while T-cells specific for EBV and CMV that establish persistent infections almost always expand, with the degree of expansion correlating with virus load [9,11]. After HSCT therefore, triVSTs expressing a first generation GD2.CAR (without co-stimulatory endodomains) should receive effective *in vivo* costimulation from professional APCs presenting antigens from endogenous EBV and CMV, and from adenovirus if the patient has such an infection. Since not all patients reactivate all viruses, increasing the number of viruses targeted by CAR-T-cells will increase the chances for stimulation.

We have previously demonstrated the clinical anti-viral efficacy of triVSTs [11]. However our manufacturing SOP comprised an initial stimulation of PBMCs using monocytes transduced with an adenoviral vector expressing pp65 of CMV (Ad5f35-pp65), while the EBV-specific component was not activated until day 9, when the cultures were restimulated with autologous EBV-LCLs transduced with Ad5f35-pp65. Therefore, this SOP required considerable modification to achieve transduction of T-cells specific for all three viruses on day 3 of culture. The simplest most effective APCs proved to be Ad5f35-pp65-transduced LCLs. LCLs are potent APCs since they express a range of costimulatory molecules, and they are excellent feeder cells that can be transduced with adenovirus type 5 provided it has a chimeric fiber with elements of Ad35 that allows transduction via CD46 [11,20]. Although the Ad vector does not express its genome, LCLs effectively process and present hexon and penton, the most abundant virion proteins, to AdVSTs, and such T-cells have been shown to control Ad infections [11,21]. CMV-pp65, expressed as a transgene is efficiently processed and presented to T-cells by transduced LCLs.

In our previous studies using gene-modified VSTs, [1,7,14,22,23] we routinely performed transduction on day 19, three days after the third stimulation, at which time VSTs were proliferating sufficiently well to produce reasonable transduction efficiencies, whereas transduction after the first stimulation produced low levels of transduction. In this work we have used the IL-4 and IL-7 for expansion of triVSTs *in vitro*, because this combination of cytokines promoted survival and expansion of VSTs without loss of specificity and induced high frequencies of viral antigen-specific IFNγ-producing T cells both in CD4^+^ and CD8^+^ T cell, at levels substantially higher than T cells generated with other cytokines, such as IL-15 and IL-2 [8]. In addition, improved media formulations (Click’s medium combined with RPMI-1640 or Advanced RPMI-1640) and the adoption of the retroviral vector centrifugation step enabled us to obtain high levels of retrovirus transduction of VSTs even with a less than optimal vector. Transduction levels of 30 to 40% were observed within a few days of transduction, when irrelevant T-cells remained in the cultures, and increased to up to 90% after antigen-driven T-cell expansion.

Our GMP compliant method for the early transduction of triVSTs providing the possibility of cryopreservation after a single stimulation is attractive as it allows for the clinical use of less differentiated VSTs and requires significantly less GMP manufacturing time. The requirement for a second stimulation is dependent on the starting PBMC number, the rate of VST expansion, the patient size and dose and the number of doses allowed. We observed an average of 5.4 ± 1.8-fold expansion after a single stimulation, so that 25 × 10^6^ PBMCs from about 25 mLs of blood could produce about 142 × 10^6^ triVSTs after 9 days in culture, more than sufficient for the proposed clinical trial (NCT01460901).

The GMP validation of our new cell production strategy yielded even higher levels of transduction than the preclinical studies. We ascribe this to the replacement of RPMI-1640 with Advanced RPMI that enhances the rate of T-cell expansion (not shown). TriVSTs cultured in this medium (45% advanced RPMI, 45% Click’s medium and 10% fetal bovine serum (FBS) produced over 80% transduction after the second stimulation and over 60% after the first stimulation in every case. Centrifugation of the retroviral vector onto the retronectin-coated plates also improved transduction and reduced the volume of vector supernatant required from 5 mLs to 2 mLs per 1 × 10^6^ T-cells. We also noted that removal of the supernatant increased the subsequent rate of T-cell expansion without loss of transduction efficiency, likely due to the removal of inhibitory factors in the vector supernatant.

## Conclusions

We developed a rapid and GMP compliant method for the early transduction of multivirus-specific T-cells that allowed stable expression of high levels of a tumor directed CAR. Perhaps the most important outcome of the optimization study was the transduction of VSTs with a central memory phenotype. Such T-cells have the potential to cycle indefinitely and repopulate both effector and memory populations, and T cells derived from this population have been shown to be more efficacious after adoptive T cell transfer [24-26]. Our earlier studies using late transduction produced lower levels of transduction of VSTs that could not be maintained during prolonged culture, although low levels of transgene could be detected years after infusion, suggesting that even when transduced late, at least some T-cells maintained memory potential. However, the true value of these improvements will be determined only by monitoring patients on the clinical trial.

## Methods

### Cells and cell lines

Blood was obtained from healthy volunteers with informed consent on a Baylor College of Medicine IRB-approved protocol and processed into PBMCs on lymphoprep (Stemcell Technologies, Vancouver, BC) gradients. PBMCs were used to manufacture EBV-LCLs (as previously described [27]) and VSTs. K562 and LAN-1 cells were obtained from the American Type Culture Collection (Rockville, MD). LCLs and K562 cells were maintained in RPMI 1640 (Hyclone, Logan, UT) with 10% fetal bovine serum (FBS; HyClone) and 2 mM L-glutamine (GlutaMAX, Invitrogen, Carlsbad, CA) while LAN-1 cells were cultured in Dulbecco’s modified Eagle’s medium (BioWhittaker, Walkersville, MD) supplemented with 10% fetal bovine serum and 2 mM L-glutamine.

### Viruses and vectors

The adenoviral vector expressing pp65 of CMV (Ad5f35-pp65) and retroviral vector, SFG.14g2a.zeta5 (GD2.CAR) encoding a first generation CAR that recognizes GD2 have been previously described [1,11]. The high-titer GFP retroviral vector was produced by co-transfection of 293 T cells with the MSCV-IRES-eGFP retroviral vector along with the gag/pol expression plasmid PegPam3(−env), the RD114 env expression plasmid RDF and MSCV vectors at a ratio of 2:3:3, respectively, using the GeneJuice transfection reagent (Calbiochem, Gibbstown, NJ) as previously described [28].

### VST Generation

To generate EBVSTs, PBMCs were stimulated with irradiated (40 Gy) autologous LCLs at a 40:1 ratio of PBMC to LCLs in T-cell media (45% RPMI 1640, Hyclone, 45% EHAA, Irvine Scientific (Santa Ana, CA), 10% heat inactivated FCS, Hyclone, and 2 mM L-glutamine, for the validation experiments in the GMP, RPMI 1640 was replaced with 45% Advanced RPMI, Invitrogen). On day 9 and weekly thereafter, EBVSTs were restimulated with irradiated LCLs at a 1:4 ratio and IL-2 (50U/ml) was added twice weekly from day 13–14 [27]. IL-4 (400U/ml) and IL-7 (10 ng/ml) both from R&D Systems (Minneapolis, MN) were added at the time of the first and second stimulations where indicated.

Two methods were compared for the generation of monocultures specific for CMV, adenovirus and EBV (triVSTs) and are described in the text and summarized in Figure 4. For the first stimulation in the Ad/PBMC + LCL method, PBMCs were adhered to plastic overnight, then transduced with Ad5f35-pp65 at the indicated virus particle to cell ratio for the indicated times at 37°C/5%CO_2_ and were then cocultured with LCLs (at the indicated ratios) for 9 days. In the Ad/LCL method, PBMCs were stimulated with Ad5f35-pp65-transduced LCL at a 40:1 ratio in T-cell medium containing 400 U/ml IL-4 and 10 ng/ml IL-7 in 24-well plates. For the second and subsequent stimulations on days 9, 16 and 23 the triVSTs were cocultured with irradiated Ad5f35-pp65-transduced LCLs at a 1:5 ratio (T cells : LCLs) in a G-Rex100 (Wilson-Wolf Manufacturing, Minneapolis, MN). On second stimulation, the VSTs received 10 ng/ml IL-7 and 400 U/ml IL-4, and then IL-2 (50U/ml) 3–4 days after the second stimulation and twice weekly thereafter.

### Transduction of triVSTs

In our previous clinical trials, EBVSTs or triVSTs were transduced on day 19 with a different method. Retroviral supernatant was adhered to retronectin-coated coated plates (0.5 mL for 30 minutes × 2), then 0.5 × 10^6^ EBVSTs were resuspended in 1.5 mL of retroviral supernatant and 0.5 mL of fresh medium and centrifuged for 5 minutes on the coated plates before culture. In the current study, the transduction was performed either early (three days after the first stimulation) or late (three days after the third stimulation on day 19). In both cases, 1.5 ml of retroviral supernatant was added per well of a retronectin (Takara, Mountain View, CA) coated, non-tissue culture-treated plates and centrifuged at 2000 × G for 1 hour at 30°C to allow virus adherence of the retronectin and removal of the possible inhibitory factors that are present in retroviral supernatants. Following removal of the retroviral supernatant, 0.5 × 10^6^ VSTs were added to each well in T-cell media containing 10 ng/ml IL-7 and 400 U/ml IL-4. The plates were centrifuged at 1000 × G for 5 minutes at room temperature and then incubated at 37°C/5%CO_2_ for an additional 7 days. Expression of the GD2.CAR was assessed by flow cytometry using the 1A7 antibody 4–5 days later and at various times during subsequent culture.

### Flow Cytometry

#### Immunophenotyping

The GD2.CAR was detected with the idiotypic antibody 1A7 [29]. To detect peptide epitope-specific T-cells in the triVSTs, we used unlabeled pentamers (Proimmune, Sarasota, FL) comprising HLA-A*0201-NLVPMVATV (from pp65 of CMV), HLA-A*0201-CLGGLLTMV (from LMP2 of EBV) and A*2401-TYFSLNNKF (from hexon of adenovirus). TriVSTs were incubated with unlabeled pentamer followed by Pro5 Fluorotag (Proimmune) in accordance with the manufacturer’s instructions. The VSTs were stained with monoclonal antibodies to CD3, CD45RO, CCR7, CD8, and CD4 (Becton Dickinson, Franklin Lakes, NJ). Approximately 20,000 events from each culture were acquired with a Gallios Flow Cytometer and analyzed with Kaluza software (Beckman Coulter, Brea, CA).

#### eFluor dilution assay

To measure T cell proliferation ET and LT triVSTs were incubated with 3 μM eFluor670 according to manufacturer’s protocol (eBioScience). One million of T cells were seeded per well of a 24-well plate with 2.5 × 10^5^ LCLs or 2.5 × 10^5^ Ad5f35pp65-transduced LCLs or 2 × 10^5^ of LAN-1 cells. T cells were stained with anti-CD3-Alexa Fluor 750, CD8-FITC, CD4-PacBlue (Beckman Coulter). Approximately 10,000 live (7AAD-negative, CD3^+^) T cells were acquired and analyzed using FlowJo software (Ashland, OR)*.*

### Elispot assay

Interferon-γ (IFNγ) release by triVSTs was assessed by enzyme-linked immunospot (ELISPOT) assay. Serial dilutions of triVSTs were plated in triplicate, starting at 10^5^ cells per well of a 96-well MultiScreen HTS IP plate (EMD Millipore, Billerica, MA), and stimulated with overlapping peptide libraries (pepmixes) (JPT Peptide Technologies) spanning the entire protein sequences of CMV-pp65, AdV-hexon and Ad-penton and autologous LCLs. ELISPOT plates were developed as previously described [20]. Plates were sent to Zellnet Consulting, New York, NY for quantification. Results are expressed as spot-forming cells per 10^5^ cells (SFCs/10^5^ cells).

### Cytotoxicity assay

A standard 6-hour ^51^chromium-release assay at effector to target (E:T) ratios of 40:1, 20:1, 10:1 and 5:1 was used to assess the cytolytic activity of transduced VSTs [22]. Target cells were GD2-positive LAN-1 cells, autologous LCLs, and K562 cells. The percent specific lysis was calculated as ([experimental release – spontaneous release]/[maximum release – spontaneous release]) × 100.

### DNA extraction and QPCR performance

DNA was extracted from transduced and non-transduced triVSTs using QIAamp DNA Blood Mini Kit (QIAGEN, Louisville, KY). QPCR was performed to determine transgene levels in transduced T cell lines (transduction efficiency). Transgene specific TaqMAN custom assays were ordered from Life Technologies, Carlsbad, CA. QPCR was performed on 7900HT QPCR machine using TaqMAN Universal Mastermix (Life Technologies, Grand Island, NY) according to manufacturer instructions. 100 ng of extracted genomic DNA was used per reaction, QPCR was performed in triplicates. Quantification was based on a standard curve prepared according to the ABI standard curve manual (Applied Biosystems). Transgene carrying plasmid dilutions were used to obtain a standard curve. The sensitivity of the assay was 1 copy per reaction.

### Culture and cryopreservation of clinical-grade VSTs

Clinical-grade VSTs were expanded in 45% advanced RPMI (Invitrogen), 45% Click’s medium (IrvineScientific, Santa Ana, CA) and 10% FBS (HyClone). Although it would be desirable to remove serum our media for the production of GMP products, we have yet to find a serum free medium that supports the growth of virus-specific T-cells. We have used highly characterized, heat-inactivated fetal calf serum for the manufacture of clinical grade T-cells that have been safely infused into hundreds of patients over the last 20 years, and since the fetal calf serum derives from American herds maintained specially for the production of fetal calf serum, there seems to be little likelihood of the transfer of unknown microbial agents. For safety reasons at the time of harvest and cryopreservation we wash cells four times to remove the traces of FBS in the formulated cell products. CAR-transduced triVSTs were cryopreserved at 1 × 10^7^ cells/mL in cryo-medium containing 12.5% Human Albumin Flexbumin USP (Baxter Healthcare Corporation, Westlake Village, CA), 10% DMSO (Bioniche Pharma USA LLC, Lake Forest, IL) and 1×HBSS (Sigma Aldrich Co, LLC, St Louis, MO) using a Cryomed 7454 programmable freezer (Thermo Fisher Scientific, Waltham, MA). Cells were recovered from storage in vapor phase liquid nitrogen and were analyzed for viability by trypan blue.

## Additional file

Additional file 1: Figure S1. GD2.CAR-transduced and non-transduced tri-VSTs from three donors were analyzed in IFN-g ELISPOT assay with titrated cognate peptides in duplicates. Serial dilutions of pepmix libraries spanning LMP2 (A) and CMV pp65 (B) proteins were used. Responses were analyzed by two-way ANOVA and statistically significant difference was found only for donor 3 at 50 and 25 ng/mL of pp65 (B), which may be a result of a technical error. **Figure S2.** Upregulation of GD2.CAR expression in CD4 + CD3+ and CD8+ CD3+ triVSTs, which were early (ET) and late transduced (LT) with GD2.CAR retrovirus upon stimulation for 48 hours with autologous LCLs and LCLs transduced with Ad-pp65. Representative results for one of three donors are shown. **Figure S3.** Cytokine predominantly produced by late transduced tri-VSTs. One million of early (ET) or late (LT) transduced tri-VSTs from two donors were stimulated with pp65 overalpping pepmix library or 1×105 autologous LCLs transduced with Ad5f35-pp65. Supernatants were collected 24 hours after the stimulation and analyzed by multiple cytokines with multiplex cytokine magnetic bead assay (EMD Millipore). **Figure S4.** Higher proliferation in response to LCLs of both CD4+ and CD8+ CD3+ T cells in early transduced (ET) cells compared to late-transduced (LT) cells. (A) Histograms are representative eFluor dilution profiles with individual divisions depicted in blue. Representative results from one of three donors are shown. Individual divisions are highlighted in blue lines and assigned by FlowJo analysis. (B) Cell division indexes were calculated using FlowJo software. Results are shown as mean ± SD, n = 3.

## Abbreviations

AdV: Adenovirus
CAR: Chimeric antigen receptor
CMV: Cytomegalovirus
EBV: Epstein-Barr virus
GMP: Good manufacturing practices
HSCT: Hematopoietic stem cell transplant
LCL: Lymphoblastoid cell line
NHLBI: National Heart, Lung and Blood Institute
PACT: Production assistance for cellular therapy
PAMPs: Pattern-associated molecular patterns
PBMC: Peripheral blood mononuclear cells
TCR: T-cell receptor
VST: Virus-specific T cells
