# Supplementary material for: Early transduction produces highly functional chimeric antigen receptor-modified virus-specific T-cells with central memory markers: a Production Assistant for Cell Therapy (PACT) translational application
Source: J Immunother Cancer. 2015 Feb 18;3:5. doi: 10.1186/s40425-015-0049-1 (PMC4346112; doi:10.1186/s40425-015-0049-1)
Supplement: Supplementary file 1 [file 40425_2015_49_MOESM1_ESM.doc]

## Slide 1

Supplementary Figure 1. Sun, et al

A.

B.

200

100

50

25

12.5

6.3

3.1

1.6

pp65, ng/mL

100

300

500

SFC/1x105 T cells

*

*

200

100

50

25

12.5

6.3

3.1

1.6

LMP2, ng/mL

100

50

SFC/1x105 T cells

Supplementary Figure 1. GD2.CAR-transduced and non-transduced tri-VSTs from three donors were analyzed in IFN-γ ELISPOT assay with titrated cognate peptides in duplicates. Serial dilutions of pepmix libraries spanning LMP2                 (A) and CMV pp65 (B) proteins were used. Responses were analyzed by two-way ANOVA and statistically significant difference was found only for donor 3 at 50 and 25 ng/mL of pp65 (B), which may be a result of a technical error. 

GD2.CAR Transduced
Non-transduced
GD2.CAR Transduced
Non-transduced
GD2.CAR Transduced
 Non-transduced
Donor 1
Donor 2
Donor 3

## Slide 2

Supplementary Figure 2. Sun, et al

Supplementary Figure 2. Upregulation of GD2.CAR expression in CD4+CD3+ and CD8+ CD3+ triVSTs, which were early (ET) and late transduced (LT) with GD2.CAR retrovirus upon stimulation for 48 hours with autologous LCLs and LCLs transduced with Ad-pp65. Representative results for one of three donors are shown.

LT

ET

CD3

CD4+

CD8+

GD2 CAR

CD4+

CD8+

Non-transduced

CD4+

CD8+

Unstim.

+LCL

+LCL
Adpp65

## Slide 3

Supplementary Figure 3. Sun, et al

LCL-Adpp65
LCL-Adpp65
no stim
pp65
no stim
pp65
donor 1
donor 2

LCL-Adpp65
LCL-Adpp65
no stim
pp65
no stim
pp65
donor 1
donor 2

LCL-Adpp65
LCL-Adpp65
no stim
pp65
no stim
pp65
donor 1
donor 2

CXCL10/IP-10

TNF-β

CCL22

Supplementary Figure 3. Cytokine predominantly produced by late transduced tri-VSTs. One million of early (ET) or late (LT)  transduced tri-VSTs from two donors were stimulated with pp65 overalpping pepmix library or 1x105 autologous LCLs transduced with Ad5f35-pp65. Supernatants were collected 24 hours after the stimulation and analyzed by multiple cytokines with multiplex cytokine magnetic bead assay (EMD Millipore). 

ET
LT

pg/mL

## Slide 4

LT
ET
eFluor 670
CD4+
CD8+
Cell count

Supplementary Figure 4. Higher proliferation in response to LCLs of both CD4+ and CD8+ CD3+ T cells in early transduced (ET) cells compared to late-transduced (LT) cells. (A) Histograms are representative eFluor dilution profiles with individual divisions depicted in blue. Representative results from one of three donors are shown.  Individual divisions are highlighted in blue lines and assigned by FlowJo analysis. (B) Cell division indexes were calculated using FlowJo software.  Results are shown as mean±SD, n=3.

Supplementary Figure 4. Sun, et al

A

B

0.5

1.0

1.5

Division index

CD4+

CD8+

P=0.0067

ET

LT
